# Identification of microRNA signature in different pediatric brain tumors

**DOI:** 10.1590/1678-4685-GMB-2016-0334

**Published:** 2018-03-26

**Authors:** Marwa Tantawy, Mariam G. Elzayat, Dina Yehia, Hala Taha

**Affiliations:** 1 Research Department, Children’s Cancer Hospital Egypt, Cairo, Egypt; 2 Pathology Department, Children’s Cancer Hospital Egypt, Cairo, Egypt; 3 Pathology Department, National Cancer Institute, Cairo University, Cairo, Egypt

**Keywords:** microRNA, pediatric, central nervous system tumors

## Abstract

Understanding pediatric brain tumor biology is essential to help on disease stratification, and to find novel markers for early diagnosis. MicroRNA (miRNA) expression has been linked to clinical outcomes and tumor biology. Here, we aimed to detect the expression of different miRNAs in different pediatric brain tumor subtypes to discover biomarkers for early detection and develop novel therapies. Expression of 82 miRNAs was detected in 120 pediatric brain tumors from fixed-formalin paraffin-embedded tissues, low-grade glioma, high-grade glioma, ependymoma, and medulloblastoma, using quantitative real-time PCR. Low-expression of miR-221, miR-9, and miR-181c/d and over-expression of miR-101, miR-222, miR-139, miR-1827, and miR-34c was found in medulloblastoma; low expression of miR-10a and over-expression of miR-10b and miR-29a in ependymoma; low expression of miR-26a and overexpression of miR-19a/b, miR-24, miR-27a, miR- 584, and miR-527 in low-grade glioma. Cox regression showed differential miRNA expression between responders and non-responders. The most specific were miR-10a and miR-29a low expression in LGG non-responders, miR-135a and miR-146b over-expression in ependymoma non-responders, and miR-135b overexpression in medulloblastoma non-responders. MicroRNAs are differentially expressed in subtypes of brain tumors suggesting that they may help diagnosis. A greater understanding of aberrant miRNA in pediatric brain tumors may support development of novel therapies.

## Introduction

Pediatric brain tumors are the second most common pediatric malignancy, representing about 25% of all childhood cancers (Boman *et al.*, 2009; Birks *et al.*, 2011). As a result of the high mortality rate and poor prognosis of brain tumors, many studies have focused on the molecular aspects of the disease, including the use of microRNAs (miRNAs) as diagnostic and prognostic markers and even more as therapeutic agents (Wang *et al.*, 2015a; Wang *et al.*, 2015b).

miRNAs are small non-coding RNAs (18–25 nucleotides) that regulate gene expression in many cellular processes by affecting the post-transcriptional regulation (Bartel and Chen, 2004; Jonas and Izaurralde, 2015). During their biogenesis, miRNAs are transcribed to form hairpin structures called pri-miRNAs; the RNase III Drosha enzyme cleaves this structure in the nucleus to form precursor miRNAs (pre-miRNAs). In the cytoplasm, RNase III Dicer enzymes cleave pre-miRNAs to produce mature miRNAs (Lund *et al.*, 2004; Van Wynsberghe *et al.*, 2011). miRNAs are unable to perform their function until the binding with RNA-induced silencing complex occurs (Diederichs and Haber, 2007).

Earlier studies reported that miRNAs have a critical role in key pathways such as cell growth, cell differentiation and apoptosis by controlling their target gene expression. The miRNAs have negative regulation of gene expression by binding the 3’ untranslated regions of mRNA of a protein-coding gene. This causes a degradation or blockage of translation of these mRNAs (Zen and Zhang, 2012). A previous study showed the significant role of miR-601 as a putative tumor suppressor gene in pediatric medulloblastoma (MED) (Braoudaki *et al.*, 2014). Other studies have shown that the inhibition of miR-21 in glioblastoma cells increase apoptosis (Faragalla *et al.*, 2012). That makes miRNAs useful biomarkers candidates for diagnosis and prognosis of pediatric brain tumors. In addition, miRNA stability in bodily fluids, functionality in several tissue types, and their capability to detect early phase disease are all useful attributes (D’Urso *et al.*, 2015; Stoicea *et al.*, 2016).

In previous studies, some miRNAs such as miR-129 were strongly down-regulated in brain tumor samples compared to normal tissue, while miR-142-5p and miR-25 were significantly upregulated in all tumor samples compared to normal tissue (Birks *et al.*, 2011). More recently, it was demonstrated that miR-19a, miR-15b and miR-106b were significantly up-regulated in MED, while miR-128, miR-299-5p, miR-138 were significantly down-regulated compared to normal control samples (Dai *et al.*, 2017). In another study, it was observed that inhibition of miR-106b can induce G1 arrest and apoptosis in MED cells (Li *et al.*, 2015). A differential expression of miR-124 in pediatric pilocytic astrocytoma was found compared to normal brain tissues (Leichter *et al.*, 2017).

The different treatments currently used, such as radiotherapy and chemotherapy, play an essential role in improving outcomes, but finding biomarkers for better diagnosis, prognosis, and management of disease progression is necessary (Tihan *et al.*, 2008; Costa *et al.*, 2011).

To investigate the importance of miRNA expression in pediatric brain tumors, low grade glioma (LGG), ependymoma (EPN), medulloblastoma (MED), and high grade glioma (HGG), their expression levels were characterized in this study using quantitative polymerase chain reaction (qPCR). The discovery of a significant profile of miRNA expression and the ability to distinguish between different histological subtypes will have a great impact on the understanding of pediatric brain tumor biology. Taking into consideration the miRNA connection with clinical outcomes, the growing information arising from laboratory research offers great promise for the advancement of diagnosis, prognosis, and therapy.

## Materials and Methods

### Patients and samples

All tumor specimens were collected retrospectively from patients undergoing surgery at the Children’s Cancer Hospital Egypt- 57357 (CCHE) from 2008 to 2015. All studies were conducted in compliance with CCHE-institutional review board regulations (CCHE-IRP #12-2014). Formalin-fixed paraffin-embedded (FFPE) specimens (n = 120) were obtained from the Pathology Department from patients diagnosed with brain tumors (34 LGG, 31 EPN, 30 MED, and 25 HGG) according to the WHO histological tumor classification (Louis *et al.*, 2007).

### RNA extraction

For FFPE samples, total RNA was isolated from 5-10 5-μm thickness tissue sections, using a miRNeasy FFPE kit (Qiagen, Hilden, Germany) according to the manufacturer’s instructions. Total RNA quantity and quality were evaluated using a spectrophotometer (Nanodrop ND-1000, Thermo Scientific, Wilmington, USA).

### Reverse transcription and quantitative polymerase chain reaction (qRCR)

Total RNA was reverse transcribed using a miScript RT kit (Qiagen). Reactions were incubated at 37 °C for 1 h followed by inactivation of the reaction by incubation at 95 °C for 10 min. For miRNA expression profiling, the primers used for qPCR were obtained from Invitrogen. One microliter of diluted RT product was used (equivalent to 10 ng) as a template in a 10-μL PCR reaction containing 1X SYBR Green master mix (Qiagen), 200 nM miRNA-specific forward primer, and 200 nM universal primer. The conditions for qPCR were as follows: 95 °C for 10 min, followed by 40 cycles of 95 °C for 15 s, 55 °C for 30 s, and 72 °C for 30 s. All qPCR reactions were performed on a QuantStudio 6 flex real-time PCR system (Applied Biosystems, Foster City, CA, USA).

The normalized relative expression levels of miRNAs were calculated using the delta cycle threshold (dCT) method, all CT values above or equal to 35 were replaced with 35 before calculating the mean of the remaining CT values. The mean CT value was calculated for each sample (ΔCT = CT sample – CT mean expression of individual miRNA plate) (Mestdagh *et al.*, 2009).

### Statistical analysis

Statistical evaluation was done using GraphPad Prism software version 5.01 (GraphPad, Inc., San Diego, CA, USA) and the SPSS win statistical package version 18. Numerical data are reported as mean ± standard deviation (SD), median, and range. Qualitative data are reported as frequency and percentage. The normal distribution of variables was assessed prior to selecting the tests for statistical analyses. The values of miRNAs were analyzed using either nonparametric Kruskal-Wallis or unpaired Student *t*-tests. The relationship between patient outcomes and miRNA expression profiles was analyzed using Mann-Whitney *U* tests. Stepwise backward multivariable logistic regression was performed. The survival rates were analyzed using log-rank analysis. A *P* value of less than 0.05 was considered significant.

## Results

### Patient samples

miRNA expression was measured using quantitative RT-PCR in 120 samples from pediatric brain tumors (34 LGG, 31 EPN, 30 MED, and 25 HGG); patients with the same disease were treated with the same protocol. The median age of LGG patients was 8.1 years, for EPN patients 3 years, for MED patients 6 years, and for HGG patients 9.7 years. The male/female ratio for LGG patients was 1:0, for EPN patients 1:1.7, for MED patients 3:1, and for HGG patients 1:3. The clinical and pathological characterization of patients enrolled in the present study is shown in Table 1.

**Table 1:** Clinicopathological features of pediatric brain tumor patients enrolled in this study.

		LGG	EPN	MED	HGG
		n = 34	n = 31	n = 30	n = 25
Age	Mean	8.6	4.9	6.7	8.7
	Median	8.1	3	6	9.7
	Range	2.8-16	0.7-16.6	2.8-14	2.1-16.6
Gender	Female	19(55.9%)	8(25.8%)	16(53.3%)	14(56%)
	Male	15(44.1%)	23(74.2%)	14(46.7%)	11(44%)
Age category	≤ 1 year	0	2(6.5%)	0	0
	> 1 year and < 10 years	23(67.6%)	24(77.4%)	25(83.3%)	16(64%)
	≥ 10 years	11(32.4%)	5(16.1%)	5(16.7%)	9(36%)
Tumor size	≤ 5 cm	21(61.8%)	15(48.4%)	21(70%)	8(32%)
	> 5 cm	12(35.3%)	15(48.4%)	8(26.7%)	14(56%)
	Unknown	1(2.9%)	1(3.2%)	1(3.3%)	3(12%)
Grade (WHO)*	I	34(100%)	0	0	0
	II	0	0	0	0
	III	0	31(100%)	0	6(24%)
	IV	0	0	30(100%)	19(76%)
Metastasis at presentation	No	34(100%)	31(100%)	18(60%)	21(84%)
	Yes	0	0	12(40%)	4(16%)
Risk	High	0	31(100%)	30(100%)	25(100%)
	Low	34(100%)	0	0	0
Patient response	Complete Remission	26(76.5%)	13(41.9%)	24(80%)	3(12%)
	Partial Remission	5(14.7%)	2(6.5%)	0	2(8%)
	No Response	0	0	1(3.3%)	2(8%)
	Progressive Disease	3(8.8%)	16(51.6%)	5(16.7)	18(72%)
Event	No (median 38 Months)	30(88.2%)	15(48.4%)	22(73.3%)	5(20%)
	Yes	4(11.8%)	16(51.6%)	8(26.7%)	20(80%)
Survival status	Dead	2(5.9%)	10(32.3%)	7(23.3%)	16(64%)
	Alive	32(94.1%)	21(67.7%)	23(76.7%)	9(36%)

LGG, Low grade glioma; EPN, Ependymoma; MED, Medulloblastoma; HGG, High grade glioma; and n, number

* Based on standard WHO (World Health Organization) classification for brain tumors.

Three-year overall survival for LGG, EPN, MED, and HGG respectively was 93.8% (95% CI 63.21-71.84), 67.7% (95% CI 50.9-72.9), 75.3% (95% CI 45.962.3), and 24.4% (95% CI 26.254). Three-year event-free survival for LGG, EPN, MED, and HGG respectively was 90.9% (95% CI 57.270), 43.3% (95% CI 3358.5), 72.6% (95% CI 4461), and 15.6% (95% CI 14.8137.2).

### Selection of miRNA for tumor tissue profiling

For our study, we selected the most significant miRNAs expressed in brain tumors compared with normal cells from previous studies. Other miRNAs were selected to distinguish between different subtypes of brain tumors and few were selected specifically for prognosis. Therefore, we identified a panel of 82 miRNAs, which are provided in Table S1.

### Profiling of 82 miRNA expression patterns in pediatric brain tumors

Most miRNAs were expressed in all subtypes with no significant change. The most significantly over-expressed miRNAs were miR-19a/b, miR-24, miR-27a, miR-584, and miR-527 in LGG, miR-10b, and miR-29a in EPN, and miR-101, miR-222, miR-139, miR-1827, and miR-34c in MED. The most significantly under-expressed miRNAs were miR-26a in LGG, miR-10a in EPN, and miR-221, miR-9, and miR-181c/d in MED. Lower dCT scores were observed in subtypes representing the higher level of expression of selected miRNAs; P values of each subtype compared with other types are shown in Tables S2-S4

### miRNAs significantly associated with patient response to treatment

To elucidate whether the pre-treatment miRNA expression profile is related with the patient’s response to chemotherapy treatment, we compared the normalized pre-treatment expression profile of the 82 miRNAs in a group of chemotherapy responders with that of non-responders in LGG and EPN groups. Patients with LGG and EPN are classified as responders and non-responders according to the following; definitions: patients with complete response (CR) were considered as responders, while patients with partial remission (PR) and progressive disease (PD) were categorized as non-responders (from Children’s Cancer Group CCG-A9952, CCG-9942 respectively, according to the roadmap treatment for LGG, EPN, MED and HGG (FiguresS1-S4). Out of the 34 LGG patients investigated in this study, 26 were classified as responders while eight were non-responders. Out of the 31 EPN patients, 13 were classified as responders while 18 were non-responders (Figures S5-S7).

**Figure 1:**
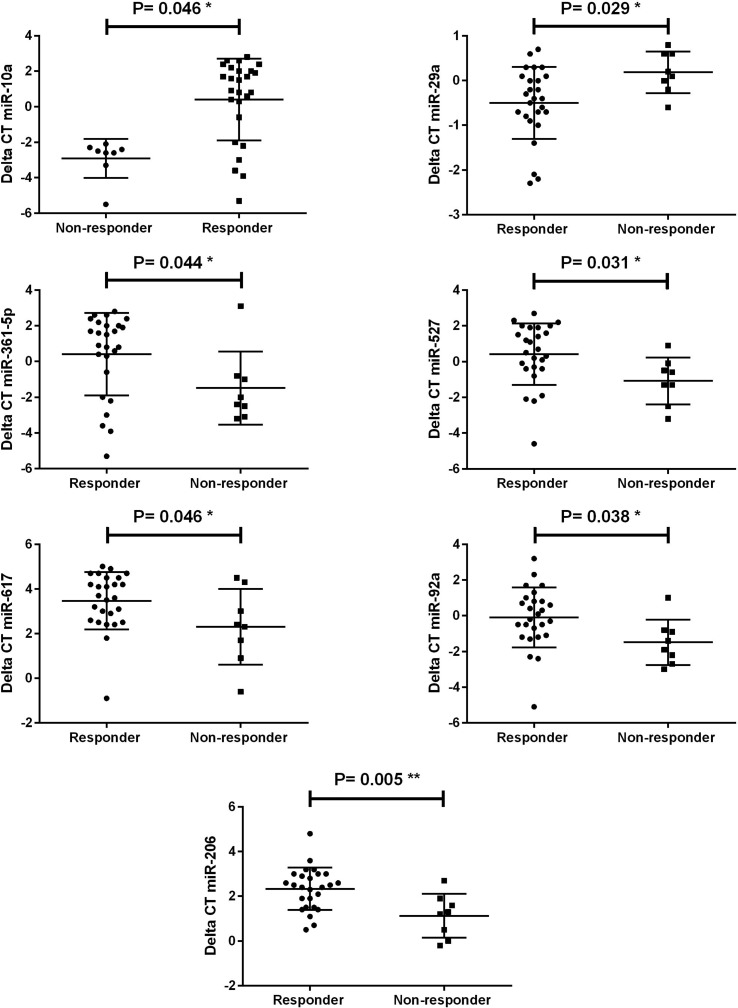
miRNAs significantly deregulated in response to treatment in LGG.

**Figure 2:**
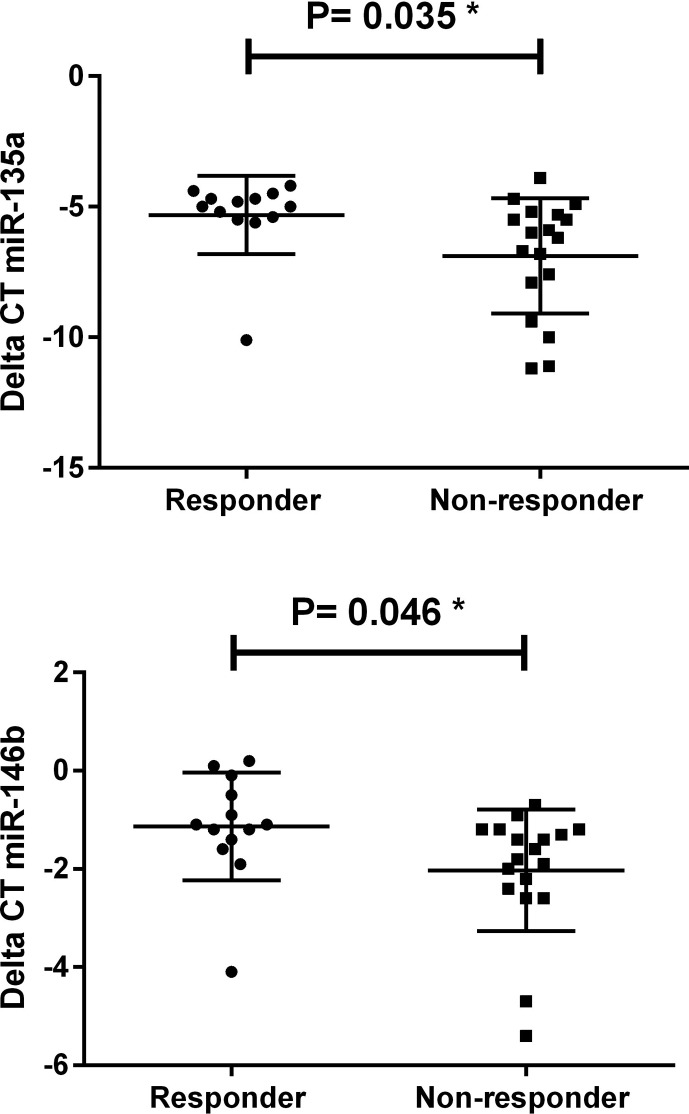
miRNAs significantly deregulated in response to treatment in EPN.

The relationship between miRNAs and treatment response was addressed by controlling for age, gender and grade using a multivariate Cox regression analysis as shown in Table 2. Low expression of miR-10a and miR-29a and high expression of miR-361-5p, miR- 617, miR-92a, miR-527, and miR-206 were detected in LGG non-responders and identified as independent factors for treatment response. We also identified miR-135a and miR-146b over-expression in EPN non-responders. The data suggest that these miRNAs could be used as biomarkers and predictors for treatment response likelihood (Figures 1 and 2).

**Table 2:** Clinicopathological features of pediatric brain tumor patients according to treatment response.

		LGG		EPN		MED		HGG	
		R	NR	R	NR	R	NR	R	NR
No.		26	8	13	18	24	6	3	22
Gender	Male	11	4	6	17	12	2	1	10
	Female	15	4	7	1	12	4	2	12
Age	≤ 1 year	0	0	1	1	0	0	0	0
	> 1 year and < 10 years	18	5	9	15	21	4	3	13
	≥ 10 years	8	3	3	2	3	2	0	9
Grade (WHO)	I	26	8	0	0	0	0	0	0
	II	0	0	0	0	0	0	0	0
	III	0	0	13	18	0	0	3	3
	IV	0	0	0	0	24	6	0	19
Tumor size	≤ 5 cm	16	5	7	8	17	4	1	7
	> 5 cm	9	3	5	10	6	2	1	13
	Unknown	1	0	1	0	1	0	1	2
Survival status	Dead	1	1	0	10	2	5	0	16
	Alive	25	7	13	8	22	1	3	6
Event	Yes	2	2	0	16	2	6	0	20
	No	24	6	13	2	22	0	3	2

LGG, Low grade glioma; EPN, Ependymoma; MED, Medulloblastoma; HGG, High grade glioma; and n, number

R, Responder; NR, Non-Responder. *Based on standard WHO (World Health Organization) classification for brain tumours.

## Discussion

miRNAs have been identified as critical regulators of tumorigenesis in a variety of cancers, but their role in pediatric brain cancers has only recently been recognized. To explore the importance of miRNAs in pediatric brain tumors, FFPE specimens of LGG, EPN, MED, and HGG were selected for miRNA expression using RT-qPCR. We identified several miRNAs expressed differentially between different pathological subtypes.

In our study, miR-10b was overexpressed in EPN compared to other subtypes. A previous study, however, showed significant alteration in the expression of miR-10b in HGG compared to LGG cell lines (Sasayama *et al.*, 2009; Visani *et al.*, 2014a). Other studies suggested that the overexpression in miR-10b in cancer cells may be correlated with an increase in hypoxia (Haque *et al.*, 2011). A study investigating predictive markers to help in glioma prognosis found that the increase in miR-10b expression in adult patients with glioma, including high and low grade gliomas, is associated with poor prognosis (Zhang *et al.*, 2016).

miR-26a was found decreased in LGG compared to other high-grade subtypes; this is in accordance with a recent study that showed that miR-26a-5p levels decreased with glioma grade (Sharma *et al.*, 2016). We observed a low expression of miR-9 in MED, in accordance with recently reported studies in MED and neuroblastoma (Laneve *et al.*, 2007; Ferretti *et al.*, 2009). In contrast with another study that reported down-regulation of miR-101 in FFPE of HGG but not in LGG (Visani *et al.*, 2014b), we found an overexpression of miR-101 in MED patients.

In our study, miR-10b was overexpressed not only in glioma tissues but also in glioma cell lines

This study showed that there are specific miRNAs in each subtype differentially expressed in responders and non-responders to chemotherapy: in LGG there was a lower expression of miR-10a in non-responders compared to responders, while previous studies have shown miR-10a regulating T follicular helper maturation (Paladini *et al.*, 2016). Significantly lower expression was also detected for miR-29a in LGG non-responders compared to responders, while a previous study identified miR-29 as a negative regulator of the B7-H3 protein, which acts as a surface immunomodulatory glycoprotein inhibiting natural killer (NK) and T-cell functions (Xu *et al.*, 2009). A previous study showed an inverse correlation between miR-29 and B7-H3 in solid tumors in cell line experiments (Wang *et al.*, 2013); another study showed the role of miR-29 in the promotion of anti-tumor immunity mediated by NK and T-cells (Filipazzi *et al.*, 2012).

In EPN patients, overexpression of miR-146a was found in non-responders compared to responders, while another study showed the role of miR-146a in inhibiting T cytotoxic immune responses (Liang *et al.*, 2015).

Significant overexpression of miR-135a and miR-135b was detected in EPN and MED in non-responders, respectively. A previous study showed that overexpression of miR-135a/b increased the resistance of lung cell lines treated with cisplatin (Zhou *et al.*, 2013).

Certain miRNAs have been correlated with outcomes of brain tumors, however these were not significant in this study. Another study found that the decrease of miR-124 may be correlated with malignant progression and poor prognosis in adult patients with gliomas (Chen *et al.*, 2015). A positive correlation was found between the overexpression of miR-219 and overall survival in pediatric patients with MED (Pezuk *et al.*, 2017). In addition, the over expression of miR-19a and miR-106b showed a significant correlation with tumor grade III of EPN (Zakrzewska *et al.*, 2016).

Additional studies with larger cohorts are needed to confirm the potential biomarkers reported in this pilot study. Pediatric brain tumors have unique miRNA profiles and the characterization of miRNA expression in serum may be an interesting follow-up study. A greater understanding of the aberrant expression of miRNAs in brain tumors of different subtypes may aid in the discovery of novel therapeutic methods.

## Supplementary material

The following online material is available for this article:

Table S1 - List of miRNA sequences used in this study.

Table S2 - dCT values of differently expressed miRNAs in MED compared to other subtypes (EPN, LGG, and HGG).

Table S3 - dCT values of differently expressed miRNAs in EPN compared to other subtypes (MED, LGG, and HGG).

Table S4 - dCT values of differently expressed miRNAs in LGG compared to other subtypes (MED, EPN, and HGG).

Figure S1 - Roadmap treatment of LGG.

Figure S2 - Roadmap treatment of EPN.

Figure S3 - Roadmap treatment of MED.

Figure S4 - Roadmap treatment of HGG.

Figure S5 - MRI images for a representative LGG case with a good therapy response.

Figure S6 - MRI images for a representative LGG, for a non-responder case.

Figure S7 - MRI images for a representative EPN case with a good therapy response case.
