# Erratum

**DOI:** 10.1590/1678-4685-GMB-2016-0334er

**Published:** 2018

In the article "Identification of microRNA signature in different pediatric brain tumors" with DOI number 10.1590/1678-4685-GMB-2016-0334, published in the journal Genetics and Molecular Biology, 41(1):27-34, on page 28 the confidence intervals stated in section Patient samples were reported wrongly.

Instead of:

Three-year overall survival for LGG, EPN, MED, and HGG respectively was 93.8% (95% CI 63.21-71.84), 67.7% (95% CI 50.9-72.9), 75.3% (95% CI 45.962.3), and 24.4% (95% CI 26.254). Three-year event-free survival for LGG, EPN, MED, and HGG respectively was 90.9% (95% CI 57.270), 43.3% (95% CI 3358.5), 72.6% (95% CI 4461), and 15.6% (95% CI 14.8137.2).

Correct is:

Three-year overall survival for LGG, EPN, MED, and HGG respectively was 93.8% (95% CI 85.57-102.03), 67.7% (95% CI 51.24-84.16), 75.3% (95% CI 59.04-91.56), and 40.7% (95% CI 20.52-60.88). Three-year event-free survival for LGG, EPN, MED, and HGG respectively was 90.9% (95% CI 81.1-100.7), 49.5% (95% CI 30.88-68.12), 72.6% (95% CI 56.34-88.86), and 28% (95% CI 10.36-45.64).

The authors informed that the correction does not affect the general results and conclusions of the study.

