# Supplementary material for: Identification of microRNA signature in different pediatric brain tumors
Source: Genet Mol Biol. 2018 Mar 26;41(1):27–34. doi: 10.1590/1678-4685-GMB-2016-0334 (PMC5901491; doi:10.1590/1678-4685-GMB-2016-0334)
Supplement: Supplementary file 1 [file 1415-4757-GMB-41-01-2016-0334-s001.pdf]

Supplementary Material to “Identification of microRNA signature in different pediatric brain tumors”

Table S1- List of miRNA sequences used in this study.

No	miRNA Name	Sequence from miRBase	miRBase Accession #	Reference	Way of comparison
1	hsa-miR-101-3p	UACAGUACUGU GAUAACUG	MIMAT0000099	(Smits *et al*. 2010)	High grade glioma compared to non-neoplastic brain
2	hsa-miR-106b-5p	AAGUGCUGACA GUGCAG	MIMAT0000680	(Birks *et al*. 2011)	Down regulated in low grade glioma compared to other subtypes in brain tumor.
3	hsa-miR-107	CAGCAUUGUAC AGGGCU	MIMAT0000104	(He *et al*. 2013b)	Down regulated in glioma.
4	hsa-miR-10a-5p	CCUGUAGAUCC GAAUUUG	MIMAT0000253	(Birks *et al*. 2011)	Down regulated in high grade glioma compared to other subtypes in brain tumor.
5	hsa-miR-10b-5p	CCUGUAGAACC GAAUUUG	MIMAT0000254	(Birks *et al*. 2011)	Down regulated in high grade glioma compared to other subtypes in brain tumor.
6	hsa-miR-1224-5p	UGAGGACUCGG GAGGU	MIMAT0005458	(Liu *et al*. 2013)	High and low grade compared to adjacent noncancerous tissues.
7	hsa-miR-124-5p	CGUGUUCACAG CGGACCUUG	MIMAT0004591	(Omran *et al*. 2013); (Wei *et al*. 2013)	Most abundant brain-specific miRNA
8	hsa-mir-1259	AUAUAUGAUG ACUUAGCUU	MIMAT0005910	(Liu *et al*. 2013)	Upregulated in high and low grade compared to adjacent noncancerous tissues.
9	hsa-miR-125a-5p	CCUGAGACCCU UUAACC	MIMAT0000443	(Fernandez-L *et al*. 2009; Birks *et al*. 2011)	Significant in medulloblastoma versus normal control.
10	hsa-miR-125b-5p	UCCCUGAGACC CUAACUUGU	MIMAT0000423	(Fernandez-L *et al*. 2009)	Significant in medulloblastoma versus normal control.
11	hsa-miR-211-5p	CCUUUGUCAUC CUUCGC	MIMAT0000268	(Asuthkar *et al*. 2012)	Significant in GBM compared to normal brain tissue.
12	hsa-miR-128-3p	UCACAGUGAAC CGGUCUC	MIMAT0000424	(Liu *et al*. 2013)	Down regulated in low and high grade compared to adjacent noncancerous tissues.
13	hsa-miR-129-5p	UUUUGCGGUCU GGGCUU	MIMAT0000242	(Birks *et al*. 2011)	Expressed in pediatric brain tumor types compared to normal tissue control.
14	hsa-miR-1296-5p	GCCCUGGCUCC AUCUC	MIMAT0005794	(Liu *et al*. 2013)	Down regulated in low and high grade glioma compared to adjacent noncancerous tissues.
15	hsa-miR-196b-5p	GGUAGUUUCCU GUUGUUG	MIMAT0001080	(Karsy *et al*. 2012)	Expressed in GBM compared to anaplastic astrocytoma and a normal brain.

No	miRNA Name	Sequence from miRBase	miRBase Accession #	Reference	Way of comparison
16	hsa-miR-132-5p	ACCGUGGCUUU CGAUUG	MIMAT0004594	(Omran *et al*. 2013)	Brain-specific
17	hsa-miR-1321	CAGGGAGGUGA AUGUGA	MIMAT0005952	(Liu *et al*. 2013)	Expressed in high and low grade glioma compared to adjacent noncancerous tissues.
18	hsa-miR-134-5p	UGUGACUGGUU GACCAG	MIMAT0000447	(Omran *et al*. 2013)	Brain-specific
19	hsa-miR-135a-5p	UAUGGCUUUUU AUUCCUAUGUG	MIMAT0000428	(Birks *et al*. 2011)	Down regulated in low grade glioma compared to other subtypes in brain tumor.
20	hsa-miR-135b-5	GGCUUUUCAUU CCUAUGUG	MIMAT0000758	(Birks *et al*. 2011)	Up-regulated in medulloblastoma and down regulated in low grade glioma compared to other subtypes in brain tumor.
21	hsa-miR-137	AUUGCUUAAGA AUACGCGU	MIMAT0000429	(Birks *et al*. 2011)	Down regulated in ependymoma compared to other subtypes in brain tumor.
22	hsa-miR-138-5p	AGCUGGUGUUG UGAAUCAGG	MIMAT0000430	(Birks *et al*. 2011; Omran *et al*. 2013; Qiu *et al*. 2013)	Down regulated in ependymoma compared to other subtypes in brain tumor.
23	hsa-miR-146a-5p	GAGAACUGAAU UCCAUGG	MIMAT0000449	(Omran *et al*. 2013)	Inflammation-related miRNAs in Astrocytes.
24	hsa-miR-146b-5p	GAGAACUGAAU UCCAUAGG	MIMAT0002809	(Birks *et al*. 2011)	Down regulated in medulloblastoma compared to other subtypes in brain tumor.
25	hsa-miR-139-5	AGUGCACGUGU CUCCAG	MIMAT0000250	(Li *et al*. 2013)	Down regulated in high grade glioma compared to normal tissue.
26	hsa-miR-181a-5p	AACAUUCAACG CUGUCGGUG	MIMAT0000256	(Omran *et al*. 2013)	Down regulated in high grade glioma compared to normal tissue.
27	hsa-miR-181b-5p	AACAUUCAUUG CUGUCGGUGG	MIMAT0000257	(Liu *et al*. 2013)	Down regulated in high grade glioma compared to normal tissue.
28	hsa-miR-181c-5p	CAUUCAACCUG UCGGUG	MIMAT0000258	(Liu *et al*. 2013)	Down regulated in high grade glioma compared to normal tissue.
29	hsa-miR-181d-5p	CAUUCAUUGUU GUCGGUG	MIMAT0002821	(Birks *et al*. 2011)	Down regulated in ependymoma compared to other subtypes in brain tumor.
30	hsa-miR-1827	UGAGGCAGUAG AUUGAAU	MIMAT0006767	(Liu *et al*. 2013)	Up-regulated in high and low grade glioma compared to adjacent noncancerous tissues.
31	hsa-miR-192-5	GACCUAUGAAU UGACAGC	MIMAT0000222	(Liu *et al*. 2013)	Down regulated in high and low grade glioma compared to adjacent noncancerous tissues.
32	hsa-miR-145-5p	GUCCAGUUUUC CCAGGA	MIMAT0000437	(Koo *et al*. 2012)	As a pro-invasive mediators in GBM. (Specific to high grade glioma).
33	hsa-miR-19a-5p	GUUUUGCAUAG UUGCACUA	MIMAT0004490	(Jia *et al*. 2013)	Up-regulated in high and low glioma compared to normal brain tissue.

No	miRNA Name	Sequence from miRBase	miRBase Accession #	Reference	Way of comparison
34	hsa-miR-19b-2-5p	UUUUGCAGGUU UGCAUUUC	MIMAT0004492	(Jia *et al*. 2013)	Up-regulated in high and low glioma compared to normal brain tissue.
35	hsa-miR-224-5p	CAAGUCACUAG UGGUUCC	MIMAT0000281	(Guo *et al*. 2015)	Specific to high grade glioma.
36	hsa-miR-373-5p	AAAUGGGGGCG CUUUCC	MIMAT0000725	(Li *et al*. 2014)	Prognostic miRNA for high grade glioma.
37	hsa-miR-203a-3p	GUGAAAUGUU UAGGACCAC	MIMAT0000264	(He *et al*. 2013a)	Specific to prognosis in glioma.
38	hsa-miR-204-5p	CCUUUGUCAUC CUAUGCC	MIMAT0000265	(Liu *et al*. 2013)	Down regulated in high and low grade glioma compared to noncancerous tissues.
39	hsa-miR-21-5p	GCUUAUCAGAC UGAUGUUG	MIMAT0000076	(Liu *et al*. 2013) (Omran *et al*. 2013)	Specific to low grade glioma compared to adjacent noncancerous tissues.
40	hsa-miR-216a-5	CUCAGCUGGCA ACUGUG	MIMAT0000273	(Birks *et al*. 2011)	Up-regulated in medulloblastoma compared to other subtypes in brain tumor.
41	hsa-miR-217	UACUGCAUCAG GAACUGA	MIMAT0000274	(Birks *et al*. 2011)	Up-regulated in medulloblastoma compared to other subtypes in brain tumor.
42	hsa-miR-218-5p	UGUGCUUGAUC UAACCAUG	MIMAT0000275	(Liu *et al*. 2013)	Down regulated in high and low grade glioma compared to adjacent noncancerous tissues.
43	hsa-miR-219a-5p	GAUUGUCCAAA CGCAAUUC	MIMAT0000276	(Rao *et al*. 2013)	Down regulated in high grade glioma compared to normal.
44	hsa-miR-22-5p	GUUCUUCAGUG GCAAGC	MIMAT0004495	(Birks *et al*. 2011)	Down regulated in medulloblastoma compared to other subtypes in brain tumor.
45	hsa-miR-221-5p	ACCUGGCAUAC AAUGUAG	MIMAT0004568	(Omran *et al*. 2013)	Inflammation-related miRNAs in astrocytes.
46	hsa-miR-222-5p	CUCAGUAGCCA GUGUAG	MIMAT0004569	(Liu *et al*. 2013; Omran *et al*. 2013)	Up- regulated in high and low grade glioma compared to adjacent noncancerous tissues.
47	hsa-miR-23a-5p	UUCCUGGGGAU GGGAUU	MIMAT0004496	(Birks *et al*. 2011)	Down regulated in medulloblastoma compared to other subtypes in brain tumor.
48	hsa-miR-24-1-5p	GCCUACUGAGC UGAUAUC	MIMAT0000079	(Liu *et al*. 2013)	Up-regulated in high and low grade glioma compared to adjacent noncancerous tissues.
49	hsa-miR-26a-5p	UUCAAGUAAUC CAGGAUAGG	MIMAT0000082	(Liu *et al*. 2013)	Down regulated in high and low grade glioma compared to adjacent noncancerous tissues.
50	hsa-miR-27a-5p	GGCUUAGCUGC UUGUGA	MIMAT0004501	(Birks *et al*. 2011)	Down regulated in medulloblastoma compared to other subtypes in brain tumor.
51	hsa-miR-297	GUAUGUGUGCA UGUGCAU	MIMAT0004450	(Kefas *et al*. 2013)	Down regulated in high grade glioma compared to normal.
52	hsa-miR-299-5p	GGUUUACCGUC CCACAU	MIMAT0002890	(Birks *et al*. 2011)	Up- regulated in low grade glioma compared to other subtypes in brain tumor.

No	miRNA Name	Sequence from miRBase	miRBase Accession #	Reference	Way of comparison
53	hsa-miR-29a-5p	CUGAUUUCUUU UGGUGUUC	MIMAT0004503	(Birks *et al*. 2011)	Up- regulated in low grade glioma compared to other subtypes in brain tumor.
54	hsa-miR-30b-5p	GUAAACAUCCU ACACUCAG	MIMAT0000420	(Fernandez-L *et al*. 2009)	Up- regulated in medulloblastoma compared to normal tissue.
55	hsa-miR-30d-5p	ACAUCCCCGAC UGGAAG	MIMAT0000245	(Fernandez-L *et al*. 2009)	Up- regulated in medulloblastoma compared to normal tissue.
56	hsa-miR-326	CUCUGGGCCCU UCCUC	MIMAT0000756	(Fernandez-L *et al*. 2009)	Down regulated in medulloblastoma compared to normal tissue.
57	hsa-miR-340-5p	UAUAAAGCAAU GAGACUGAU	MIMAT0004692	(Birks *et al*. 2011)	Up-regulated in medulloblastoma compared to other subtypes in brain tumor.
58	hsa-miR-34a-5p	GGCAGUGUCUU AGCUGG	MIMAT0000255	(Birks *et al*. 2011)	Up-regulated in low grade glioma compared to other subtypes in brain tumor.
59	hsa-miR-152-3p	UCAGUGCAUGA CAGAACU	MIMAT0000438	(Zheng *et al*. 2013)	Down regulated in glioma compared to normal.
60	hsa-miR-34c-5p	GGCAGUGUAGU UAGCUG	MIMAT0000686	(Birks *et al*. 2011)	Up-regulated in Ependymoma and down regulated in high grade glioma compared to other subtypes in brain tumor.
61	hsa-miR-361-5p	UCAGAAUCUCC AGGGGU	MIMAT0000703	(Liu *et al*. 2013)	Up-regulated in high and low grade glioma compared to adjacent noncancerous tissues.
62	hsa-miR-423-5p	GGGCAGAGAGC GAGAC	MIMAT0004748	(Liu *et al*. 2013)	Up-regulated in high and low grade glioma compared to adjacent noncancerous tissues.
63	hsa-miR-432-5p	UGGAGUAGGUC AUUGGG	MIMAT0002814	(Birks *et al*. 2011)	Up-regulated in low grade glioma compared to other subtypes in brain tumor.
64	hsa-miR-483-5p	AGACGGGAGGA AAGAAG	MIMAT0004761	(Birks *et al*. 2011)	Up-regulated in ependymoma to other subtypes in brain tumor.
65	hsa-miR-484	UCAGGCUCAGU CCCCU	MIMAT0002174	(Liu *et al*. 2013)	Down regulated in high and low grade glioma compared to adjacent noncancerous tissues.
66	hsa-miR-486-5p	UCCUGUACUGA GCUGCCCC	MIMAT0002177	(Delfino *et al*. 2011)	Specific in high grade glioma.
67	hsa-miR-191-5p	CGGAAUCCCAA AAGCAG	MIMAT0000440	(Li *et al*. 2014)	Specific to prognosis in high grade glioma.
68	hsa-miR-513b-5p	UUCACAAGGAG GUGUCAU	MIMAT0005788	(Liu *et al*. 2013)	Up-regulated in high and low grade glioma compared to adjacent noncancerous tissues.
69	hsa-miR-527	UGCAAAGGGAA GCCCUU	MIMAT0002862	(Birks *et al*. 2011)	Up-regulated in ependymoma compared to other subtypes in brain tumor.
70	hsa-miR-584-5p	GGUUUGCCUGG GACUG	MIMAT0003249	(Birks *et al*. 2011)	Up-regulated in ependymoma compared to other subtypes in brain tumor.

No	miRNA Name	Sequence from miRBase	miRBase Accession #	Reference	Way of comparison
71	hsa-miR-15b-5p	GCAGCACAUCA UGGUUUA	MIMAT0000417	(Karsy *et al*. 2012)	Significance to high grade glioma.
72	hsa-miR-617	UUCCCAUUUGA AGGUGGC	MIMAT0003286	(Birks *et al*. 2011)	Up-regulated in high grade glioma compared to other subtypes in brain tumor.
73	hsa-miR-760	CGGCUCUGGGU CUGUG	MIMAT0004957	(Liu *et al*. 2013)	Up-regulated in high and low grade glioma compared to adjacent noncancerous tissues.
74	hsa-miR-885-5p	CCAUUACACUA CCCUGC	MIMAT0004947	(Liu *et al*. 2013)	Down regulated in high and low grade glioma compared to adjacent noncancerous tissues.
75	hsa-miR-9-5p	UCUUUGGUUAU CUAGCUGUAU	MIMAT0000441	(Fernandez-L *et al*. 2009)	Down regulated in medulloblastoma compared to normal tissue.
76	hsa-miR-92a-1-5p	GUUGGGAUCGG UUGCAA	MIMAT0004507	(Baraniskin *et al*. 2011)	Specific to brain tumor.
77	hsa-miR-92b-5p	ACGGGACGCGG UGCA	MIMAT004792	(Birks *et al*. 2011; Liu *et al*. 2013; Wu *et al*. 2013)	Down regulated in medulloblastoma compared to other subtypes in brain tumor.
78	hsa-miR-93-5p	CAAAGUGCUGU UCGUGC	MIMAT0000093	(Birks *et al*. 2011)	Up-regulated in ependymoma compared to other subtypes in brain tumor.
79	hsa-miR-149-5p	UCUGGCUCCGU GUCUUC	MIMAT0000450	(She *et al*. 2014)	Down regulated in high grade glioma compared to normal brain tissue.
80	hsa-miR-143-5p	GCAGUGCUGCA UCUCUG	MIMAT0004599	(Koo *et al*. 2012)	As a pro-invasive mediators in GBM.(specific to high grade glioma)
81	hsa-miR-7-5p	UGGAAGACUAG UGAUUUUG	MIMAT0000252	(Liu *et al*. 2014)	Compared to normal brain capillaries.
82	hsa-miR-206	GAAUGUAAGG AAGUGUGUG	MIMAT0000462	(Wang *et al*. 2014)	Down regulated in glioma compared to normal brain tissue.

References

Asuthkar S, Velpula KK, Chetty C, Gorantla B and Rao JS (2012) Epigenetictegulation of miRNA-211 by MMP-9 governs glioma cell apoptosis, chemosensitivity andradiosensitivity. Oncotarget 3:1439-1454.

Baraniskin A, Kuhnhenn J, Schlegel U, Chan A, Deckert M, Gold R, Maghnouj A, Zöllner H, Reinacher-Schick A, Schmiegel W, *et al*. (2011) Identification of microRNAs in the cerebrospinal fluid as marker for primary diffuse large B-cell lymphoma of the central nervous system. Blood 117:3140-31466.

Birks DK, Barton VN, Donson AM, Handler MH, Vibhakar R and Foreman NK (2011) Survey of microRNA expression in pediatric brain tumors. Pediatr Blood Cancer 56:211-216.

Delfino KR, Serão NVL, Southey BR and Rodriguez-Zas SL (2011) Therapy-, gender- and race-specific microRNA markers, target genes and networks related to glioblastoma recurrence and survival. Cancer Genomics Proteomics 8:173-183.

Fernandez-L A, Northcott PA, Taylor MD and Kenney AM (2009) Normal and oncogenic roles for microRNAs in the developing brain. Cell Cycle 8:4049-4054.

Guo X, Xue H, Guo X, Gao X, Xu S, Yan S, Han X, Li T, Shen J and Li G (2015) MiR224-3p inhibits hypoxia-induced autophagy by targeting autophagy-related genes in human glioblastoma cells. Oncotarget 6:41620-41637.

He J, Deng Y, Yang G and Xie W (2013a) MicroRNA-203 down-regulation is associated with unfavorable prognosis in human glioma. J Surg Oncol 108:121-125.

He J, Zhang W, Zhou Q, Zhao T, Song Y, Chai L and Li Y (2013b) Low-expression of microRNA-107 inhibits cell apoptosis in glioma by upregulation of SALL4. Int J Biochem Cell Biol 45:1962-1973.

Jia Z, Wang K, Zhang A, Wang G, Kang C, Han L and Pu P (2013) miR-19a and miR-19b overexpression in gliomas. Pathol Oncol Res 19:847-853.

Karsy M, Arslan E and Moy F (2012) Current progress on understanding microRNAs in glioblastoma multiforme. Genes Cancer 3:3-15.

Kefas B, Floyd DH, Comeau L, Frisbee A, Dominguez C, Dipierro CG, Guessous F, Abounader R and Purow B (2013) A miR-297/hypoxia/DGK-α axis regulating glioblastoma survival. Neuro Oncol 15:1652-1663.

Koo S, Martin GS, Schulz KJ, Ronck M and Toussaint LG (2012) Serial selection for invasiveness increases expression of miR-143/miR-145 in glioblastoma cell lines. BMC Cancer 12:143.

Li R-Y, Chen L-C, Zhang H-Y, Du W-Z, Feng Y, Wang H-B, Wen J-Q, Liu X, Li X-F, Sun Y, *et al*. (2013) MiR-139 inhibits Mcl-1 expression and potentiates TMZ-induced apoptosis in glioma. CNS Neurosci Ther 19:477-483.

Li R, Gao K, Luo H, Wang X, Shi Y, Dong Q, Luan W and You Y (2014) Identification of intrinsic subtype-specific prognostic microRNAs in primary glioblastoma. J Exp Clin Cancer Res 33:9.

Liu F, Xiong Y, Zhao Y, Tao L, Zhang Z, Zhang H, Liu Y, Feng G, Li B, He L, *et al*. (2013) Identification of aberrant microRNA expression pattern in pediatric gliomas by microarray. Diagn Pathol 8:158.

Liu Z, Liu Y, Li L, Xu Z, Bi B, Wang Y and Li JY (2014) MiR-7-5p is frequently downregulated in glioblastoma microvasculature and inhibits vascular endothelial cell proliferation by targeting RAF1. Tumour Biol 35:10177-10184.

Omran A, Ashhab MU, Gan N, Kong H, Peng J and Yin F (2013) Effects of MRP8, LPS, and Lenalidomide on the expressions of TNF-α, brain-enriched, and inflammation-related microRNAs in the primary astrocyte culture. Sci World J 2013:208309.

Qiu S, Huang D, Yin D, Li F, Li X, Kung H-F and Peng Y (2013) Suppression of tumorigenicity by microRNA-138 through inhibition of EZH2-CDK4/6-pRb-

E2F1 signal loop in glioblastoma multiforme. Biochim Biophys Acta 1832:1697-1707.

Rao SAM, Arimappamagan A, Pandey P, Santosh V, Hegde AS, Chandramouli BA and Somasundaram K (2013) miR-219-5p inhibits receptor tyrosine kinase pathway by targeting EGFR in glioblastoma. PLoS One 8:e63164.

She X, Yu Z, Cui Y, Lei Q, Wang Z, Xu G, Xiang J, Wu M and Li G (2014) miR-128 and miR-49 enhance the chemosensitivity of temozolomide by Rap1B-mediated cytoskeletal remodeling in glioblastoma. Oncol Rep 32:957-964.

Smits M, Nilsson J, Mir SE, Van der Stoop PM, Hulleman E, Niers JM, Hamer PCDW, Marquez VE, Cloos J, Krichevsky AM, *et al*. (2010) miR-101 is down-regulated in glioblastoma resulting in EZH2- induced proliferation, migration, and angiogenesis. Oncotarget.1 710-20.

Wang S, Lu S, Geng S, Ma S, Liang Z and Jiao B (2014) Decreased expression of microRNA-206 correlates with poor clinical outcome in patients with malignant astrocytomas. Pathol Oncol Res 20:343-348.

Wei J, Wang F, Kong L-Y, Xu S, Doucette T, Ferguson SD, Yang Y, McEnery K, Jethwa K, Gjyshi O, *et al*. (2013) miR-124 inhibits STAT3 signaling to enhance T cell-mediated immune clearance of glioma. Cancer Res 73:3913-3926.

Wu ZB, Cai L, Lin SJ, Lu JL, Yao Y and Zhou LF (2013) The miR-92b functions as a potential oncogene by targeting on Smad3 in glioblastomas. Brain Res 1529:16-25.

Zheng X, Chopp M, Lu Y, Buller B and Jiang F (2013) MiR-15b and miR-152 reduce glioma cell invasion and angiogenesis via NRP-2 and MMP-3. Cancer Lett 329:146-154.
