# Supplementary material for: Identification of microRNA signature in different pediatric brain tumors
Source: Genet Mol Biol. 2018 Mar 26;41(1):27–34. doi: 10.1590/1678-4685-GMB-2016-0334 (PMC5901491; doi:10.1590/1678-4685-GMB-2016-0334)
Supplement: Supplementary file 2 [file 1415-4757-GMB-41-01-2016-0334-s002.pdf]

Supplementary Material to "Identification of microRNA signature in different pediatric brain tumors"

Table S2 - dCT values of differently expressed miRNAs in MED compared to other subtypes (EPN, LGG, and HGG).

miRNA	MED		EPN		P value	LGG		P value	HGG		P value
	Average	SD	Average	SD		Average	SD		Average	SD	
**miR-101**	-4.18	1.42	-2.30	1.70	0	-2.25	2.05	0	-2.47	1.61	0
**miR-222**	-1.97	1.04	-1.00	1.22	0	-1.03	0.85	0	-1.04	0.92	0.001
**miR-139**	-3.75	1.69	-1.85	1.58	0	-1.67	1.67	0	-1.80	0.93	0
**miR-1827**	-2.32	1.01	-1.34	1.28	0.009	-1.55	0.90	0.013	-1.27	1.12	0.004
**miR-34c**	-0.46	0.61	0.24	0.85	0.003	0.69	1.10	0	0.25	0.76	0.003
**miR-221**	-3.25	0.66	-4.29	1.47	0.005	-4.06	1.22	0.008	-4.43	1.46	0.004
**miR-9**	-0.67	2.23	-2.71	1.63	0.001	-3.43	1.86	0	-3.07	3.26	0
**miR-181d**	-3.29	0.67	-4.42	1.48	0.002	-4.11	1.18	0.006	-4.39	1.55	0.014
**miR-181c**	-2.14	0.81	-2.68	0.90	0.016	-2.66	0.99	0.019	-2.73	0.73	0.013
