# Supplementary material for: Identification of microRNA signature in different pediatric brain tumors
Source: Genet Mol Biol. 2018 Mar 26;41(1):27–34. doi: 10.1590/1678-4685-GMB-2016-0334 (PMC5901491; doi:10.1590/1678-4685-GMB-2016-0334)
Supplement: Supplementary file 3 [file 1415-4757-GMB-41-01-2016-0334-s004.pdf]

Supplementary Material to "Identification of microRNA signature in different pediatric brain tumors"

Table S4 - dCT values of differently expressed miRNAs in LGG compared to other subtypes (MED, EPN, and HGG).

miRNA	LGG		EPN		P value	MED		P value	HGG		P value
	Average	SD	Average	SD		Average	SD		Average	SD	
**miR-19a**	-1.82	2.57	0.62	3.12	0.007	1.85	0.98	0	2.02	0.81	0
**miR-19b**	-1.62	0.91	-1.05	0.64	0.03	-0.71	1.49	0.034	-0.82	1.05	0.022
**miR-24**	0.17	3.15	2.47	3.02	0.023	4.03	1.03	0	3.79	1.27	0
**miR-27a**	-0.29	3.24	2.61	3.19	0.003	3.82	2.26	0	3.15	4.30	0.01
**miR-584**	-3.33	1.54	0.76	2.59	0	-0.37	1.49	0	0.05	1.80	0
**miR-527**	0.06	1.73	1.69	1.00	0	1.34	1.00	0	1.12	1.04	0.002
**miR-26a**	-1.41	2.00	-2.65	1.57	0.04	-3.59	1.35	0	-3.44	1.32	0

LGG, Low grade glioma; EPN, Ependymoma; MED, Medulloblastoma; HGG, High grade glioma; and SD, Standard Deviation.
