# Supplementary material for: Identification of microRNA signature in different pediatric brain tumors
Source: Genet Mol Biol. 2018 Mar 26;41(1):27–34. doi: 10.1590/1678-4685-GMB-2016-0334 (PMC5901491; doi:10.1590/1678-4685-GMB-2016-0334)
Supplement: Supplementary file 4 [file 1415-4757-GMB-41-01-2016-0334-s005.pdf]

Supplementary Material to "Identification of microRNA signature in different pediatric brain tumors"

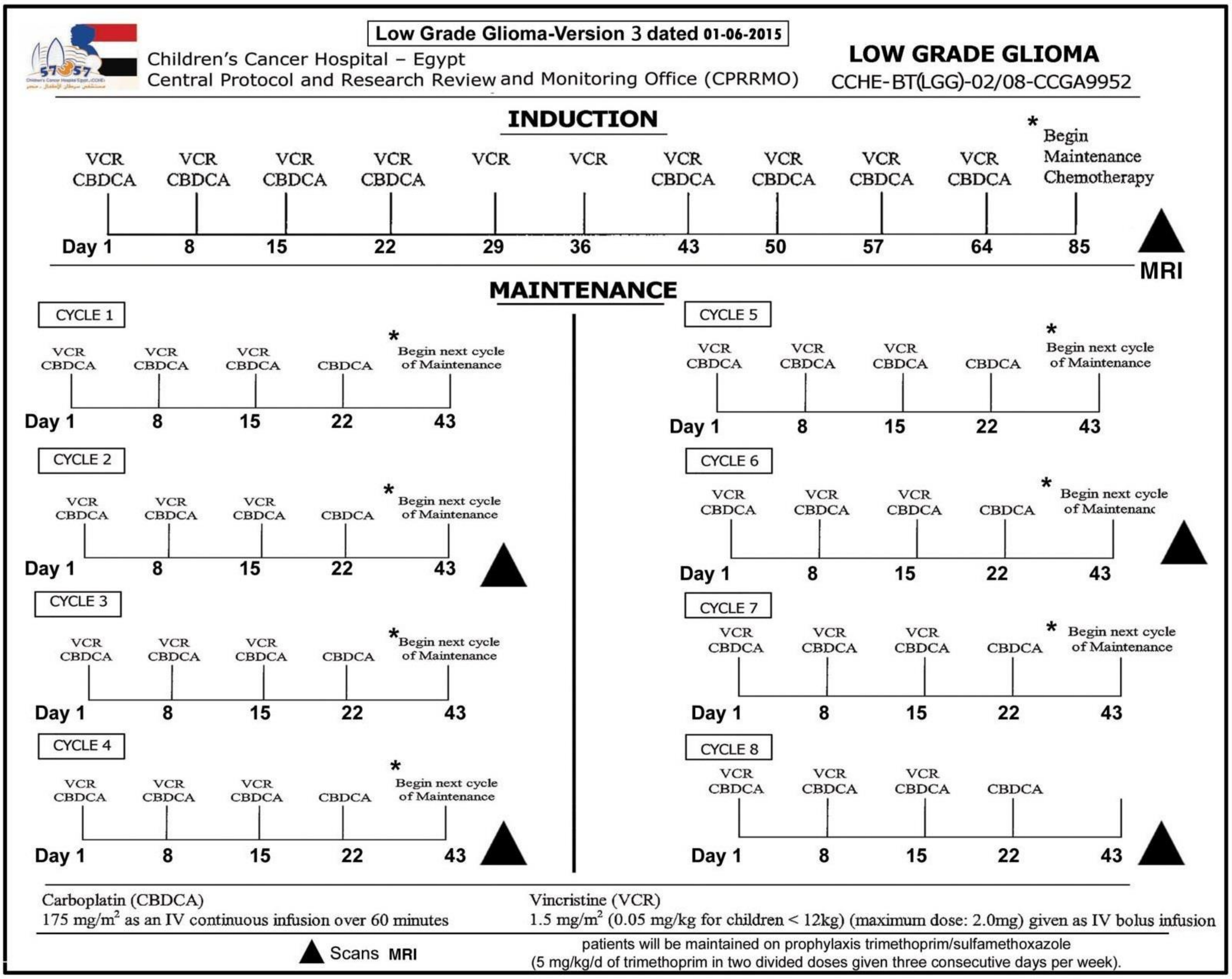

Figure S1 - Roadmap treatment of LGG.
* At the beginning of each cycle: ANC ≥ 1000 – PLT ≥ 100,000
During cycle: ANC ≥ **500**-PLT ≥ **50,000** (otherwise check protocol for dose modification)
