# Supplementary material for: Identification of microRNA signature in different pediatric brain tumors
Source: Genet Mol Biol. 2018 Mar 26;41(1):27–34. doi: 10.1590/1678-4685-GMB-2016-0334 (PMC5901491; doi:10.1590/1678-4685-GMB-2016-0334)
Supplement: Table S3 [file 1415-4757-GMB-41-01-2016-0334-s003.pdf]

Supplementary Material to "Identification of microRNA signature in different pediatric brain tumors"

Table S3 - dCT values of differently expressed miRNAs in EPN compared to other subtypes (MED, LGG, and HGG).

**miRNA**	**EPN**		**MED**		**P value**	**LGG**		**P value**	**HGG**		**P value**
	Average	SD	Average	SD		Average	SD		Average	SD	
**miR-10b**	-3.61	0.85	-2.09	1.50	0	-2.29	1.23	0	-2.11	2.42	0.036
**miR-29a**	-0.97	0.85	-0.12	0.87	0.002	-0.34	0.79	0.017	-0.22	0.86	0.011
**miR-10a**	-1.39	1.42	-2.45	0.56	0.002	-2.28	1.05	0.038	-2.48	0.77	0.004

LGG, Low grade glioma; EPN, Ependymoma; MED, Medulloblastoma; HGG, High grade glioma; and SD, Standard Deviation.
